# Improvement of Muscle Quality of Grass Carp (*Ctenopharyngodon idellus*) With a Bio-Floating Bed in Culture Ponds

**DOI:** 10.3389/fphys.2019.00683

**Published:** 2019-05-31

**Authors:** Xi Zhang, Jingwei Wang, Rong Tang, Xugang He, Li Li, Yasuaki Takagi, Dapeng Li

**Affiliations:** ^1^National Demonstration Center for Experimental Aquaculture Education, Hubei Provincial Engineering Laboratory for Pond Aquaculture, College of Fisheries, Huazhong Agricultural University, Wuhan, China; ^2^Faculty of Fisheries Sciences, Hokkaido University, Sapporo, Japan

**Keywords:** fish, muscle quality, antioxidant capacity, texture profiles analysis, water environment

## Abstract

Muscle quality and the physiological condition of fish are affected greatly by the culture environment. In aquaculture ponds, a bio-floating bed planted with vegetation is often used to improve water quality. This study investigated the growth and muscle quality of grass carp (*Ctenopharyngodon idellus*) cultured in ponds equipped with bio-floating beds. Fish were cultured in two replicated pond groups from May to November. Fish in the first group were cultured in experimental ponds equipped with a bio-floating bed planted with *Ipomoea aquatica*, whereas fish in the other group were reared in control ponds without a bio-floating bed. Compared with control ponds, the experimental ponds had better water quality with significantly lower concentrations of nitrite and ammonia. Grass carp in the experimental group had greater muscle weight gain, a significantly higher content of crude protein, and a significantly lower crude fat level than fish in the control group. The levels of pH, water-holding capacity, and antioxidant capacity of muscle decreased significantly in the control group compared to the experimental group. Texture profile analysis revealed higher values of hardness, springiness, gumminess, and chewiness and lower values of cohesiveness and resilience of white muscle in the experimental group compared to the control group. The filets of fish in the experimental group also received higher grades in the sensory evaluation of springiness, overall acceptability, aroma, and palatability. These results indicate that growth performance and muscle quality of grass carp were improved by the presence of bio-floating beds in the culture ponds.

## Introduction

Fish growth and muscle quality are affected by external and internal factors, including culture environment, food nutrition, and genetics (Johnston, 2001; Gutierrez et al., 2014; Gisbert et al., 2016; Zhao et al., 2018). Fish quality has been defined as a combination of characteristics such as wholesomeness, freshness, and integrity (Martin, 1998). Freshness, a factor that is crucial to the consumer, is reflected by appearance, taste, and texture of muscle. In addition, nutritional composition and water-holding capacity (WHC) have significant impacts on the quality of muscle. These quality parameters are influenced by intrinsic (fish species, size, and sexual maturity) and extrinsic (source of nutrients, season, water salinity, temperature) factors, and nutritional value and sensory characteristics of fish muscle are especially affected by culture conditions (Björnsson and Ólafsdóttir, 2006; Cheng et al., 2014). Consumers generally prefer wild-caught fish because of their superior organoleptic qualities, firmer texture, and better flavor relative to cultured fish (Grigorakis et al., 2003; Periago et al., 2005; Fuentes et al., 2010; Stejskal et al., 2011). Thus, how to produce cultured fish of high quality is a key concern for the aquaculture industry (Valente et al., 2013; Zhao et al., 2018).

Pond culture is the most important aquaculture model in China. However, water contamination, which negatively impacts the water environment and fish quality, has become a serious problem that limits sustainable development of pond culture systems (Björnsson and Ólafsdóttir, 2006; Qin, 2010). To address this problem, bio-floating bed technology has been used in recent years as an ecological remediation method to control water eutrophication. This low-cost, solar-energy-based, and eco-friendly technology has been used for water purification around the world (Wen and Recknagel, 2002; Todd et al., 2003; Garbett, 2005; Nduvamana et al., 2007; Nakai et al., 2008; Stewart et al., 2008). For example a floating-bed planted with *Ipomoea aquatica* was used in ponds to improve water quality, and significant reductions in nitrite and ammonium concentrations were achieved (Gao and Sun, 2008). *Ipomoea aquatica* is an aquatic macrophyte that can remove nutrients such as nitrogen and phosphorus and metals such as cadmium and chromium from the water and reduce chemical oxygen demand (COD), biological oxygen demand (BOD), and total suspended solids (TSS) (Hu et al., 2008; Wang et al., 2008; Chen et al., 2010; Chanu and Gupta, 2016).

The grass carp (*Ctenopharyngodon idellus*) is a popular fish species in freshwater aquaculture (Xie et al., 2018). However, the muscle quality of cultured grass carp has declined in recent years due to the use of artificial feeds and to the contamination of the water environment (Fuentes et al., 2010; Stejskal et al., 2011; Zhao et al., 2018). As water quality is a key factor affecting growth performance of fish (Johnston, 2001; Björnsson and Ólafsdóttir, 2006), addition of a bio-floating bed to culture ponds could purify the eutrophic water (Li et al., 2010). Thus, this technology is a promising way to improve the aquatic culture environment and would be likely to have positive effects on fish quality.

Water environment restoration can contribute to better muscle quality of fish in aquaculture systems (Johnston, 2001; Orban et al., 2002; Björnsson and Ólafsdóttir, 2006). Therefore, we hypothesized that the growth performance and muscle quality of cultured grass carp would be enhanced by using a bio-floating bed in aquaculture ponds. The purpose of this study was to investigate the growth and muscle quality of grass carp cultured in ponds equipped with bio-floating beds planted with *I. aquatica*. Results of this study will add to our understanding of how different aquaculture environments affect the characteristics of grass carp muscle quality and can be applied to improving fish quality by implementing bio-floating bed technology.

## Materials and Methods

### Experimental Protocol

**FIGURE 1:**
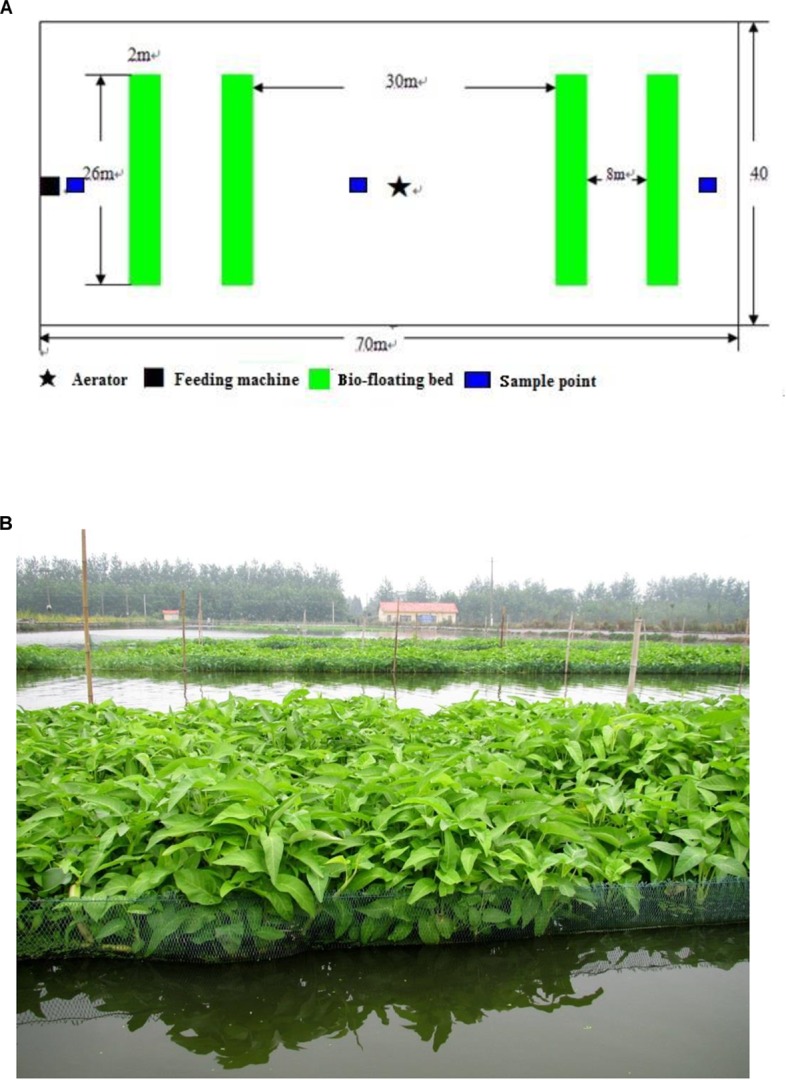
Schematic diagram of equipping the bio-floating bed in the pond **(A)** and the scenery of bio-floating bed planted with *Ipomoea aquatica* **(B)**.

This experimental protocol was approved by the Institutional Animal Care and Use Committees of Huazhong Agricultural University, China. Five aquaculture ponds (each 70 m long × 40 m wide) were used in this study, which took place from May to November at the Field Scientific Observation and Research Station of Pond Culture, Huazhong Agricultural University. Three ponds with bio-floating beds were used as the experimental pond group, and the other two without a bio-floating bed were used as the control group. The bio-floating beds were set up on the water surface of the experimental ponds (Figure 1). Each experimental pond contained four bio-floating beds that together covered 7.5% of the water surface area. After installation, each bio-floating bed was planted with *I. aquatica* with 20 cm distance between and within rows. Grass carp was the major cultured species (size, 60 g/tail) in the pond. Table 1 lists the fish species and their numbers in the five ponds. The fish density was the same in both the experimental and control ponds. The fish were fed to satiation with identical feeds (Haid Group Co., Ltd., China) containing crude protein (28.00%), crude fat (7.06%), moisture (8.75%), crude fiber (15.00%), and ash (15.63%).

**Table 1:** Fish species, size, and amount cultured in one pond.

Species	Size (g)	Amount (kg)
*Ctenopharyngodon idellus*	60	190
	400	40
*Hypophthalmichthys molitrix*	1460	50
	700	12.5
*Aristichthys nobilis*	100	8
	1060	40


### Water Quality Measurements

Water samples were collected from each pond every month during the experimental period. All water samples were obtained by mixing samples from upper, middle, and lower water layers. Each 200-mL water sample was filtered (Whatman GF/C glass-fiber 0.45 μm pore size) and analyzed for ammonium nitrogen (NH_4_^+^-N), nitrite nitrogen (NO_2_^-^-N), nitrate nitrogen (NO_3_^-^-N), and phosphate (PO_4_^3-^-P). Unfiltered subsamples were analyzed for total nitrogen (TN) and total phosphorus (TP). NH_4_^+^-N, NO_2_^-^-N, NO_3_^-^-N, and TP contents were measured using the Nesslerization colorimetric (GB/T7479-1987), diazonium coupled spectrophotometric (GB/T7493-1987), UV spectrophotometric (HJ/T346-2007), and molybdate spectrophotometric (GB/T11893-1989) methods, respectively. PO_4_^3-^-P was measured according to the method of the Editorial Board of Monitoring and Analytical Methods of Water and Wastewater (2002).

**FIGURE 2:**
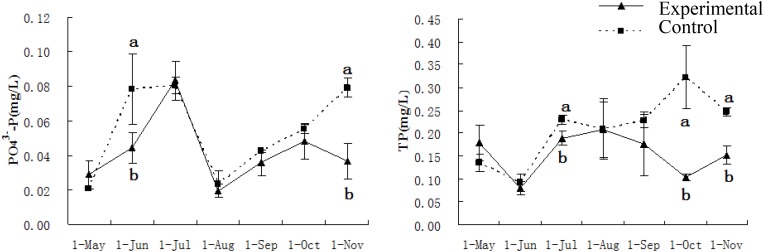
Water quality parameters of PO_4_^3-^-P and TP of experimental and control ponds. Chemical elements PO_4_^3-^-P and total phosphorus (TP) were tested in every month from May to November. Vertical bars indicate significant deviation. Meanwhile, letters of a and b indicate the significant difference (*P* < 0.05).

**FIGURE 3:**
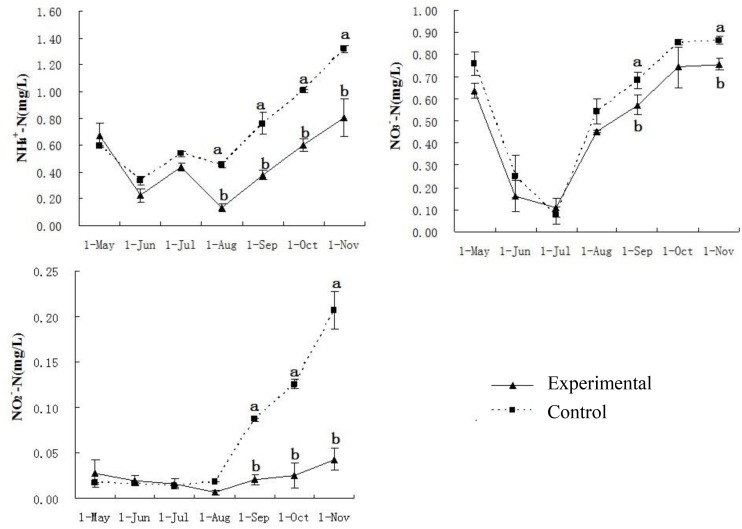
Water quality parameters of nitrogen of experimental and control ponds. Chemical elements of three various forms of nitrogen (NH_3_/NH_4_^+^, NO_3_^-^, NO_2_^-^) were tested in every month from May to November. Vertical bars indicate significant deviation. Meanwhile, letters of a and b indicate the significant difference (*P* < 0.05).

The PO_4_^3-^-P concentrations in experimental ponds were significantly lower than those of the control ponds from June and November. The concentrations of TP in the experimental ponds were significantly lower than those of the control ponds in July, October, and November (Figure 2). There were no differences in the values of the nitrogen parameters among all ponds from May to July. However, from August to November, the NH_4_^+^-N values of the experimental ponds were significantly lower than those of the control ponds. The concentrations of either NO_2_^-^-N or NO_3_^-^-N were significantly lower in the experimental ponds than in the control ponds in the late experimental period (Figure 3).

### Biometric Measurements

Twenty grass carp from each pond were sampled, and the fish were anesthetized with MS-222 (50 mg/L). Fish were individually weighed to obtain total weight (TW) and measured to obtain standard length (SL), height (H), and width (W). Condition factor (CF) was calculated as CF = (TW/SL^3^) × 100. Multiples of weight gain was calculated as final weight/initial weight. Liver and visceral mass were weighed to obtain liver weight (LW) and visceral mass weight (VW), and the liver somatic index and the visceral mass ratio were calculated as (LW/TW) × 100 and (VW/TW) × 100, respectively. Total muscle (MW) was weighed, and the flesh content was calculated as (MW/TW) × 100.

### Chemical Composition of Muscle

Dorsal white muscles of fish were sampled and kept frozen (-20°C) for chemical analysis. Chemical composition of muscle was measured according to methods provided by the National Food Safety Standard of P. R. of China. Water content was determined by method GB 5009.3-2010; ash by method GB 5009.3-2010; crude protein by the Kjeldahl method (GB 5009.5-2010), and lipid by the Soxhlet method (GB/T 5009.6-2003).

### pH and Water-Holding Capacity (WHC)

Muscle pH was measured using a Testo 205 pH meter (Testo AG, Lenzkirch, Germany). WHC of the samples was measured by drip loss, stored loss, frozen leakage rate, and cooked rate. In order to eliminate the effects of different parts of the muscle on the WHC, we used 5 g of muscle taken from the same location of each fish for the analyses. To calculate drip loss, we placed the muscle in a plastic bag hanging for 48 h at 4°C and weighed it again. To calculate the stored loss, the muscle was placed in a tightly sealed plastic bag, stored at 4°C for 24 h, and weighed again. To obtain the frozen leakage rate, muscle was kept for 24 h at -20°C, tightly sealed in a plastic bag at 4°C, and weight at 0, 1, and 2 h. The cooked rate was calculated by weighing the muscle after it was cooked for 15 min.

### Antioxidant Assays

Total antioxidant capacity (T-AOC), activities of superoxide dismutase (SOD), lipid peroxidation (LPO), and protein carbonylation (PC) were measured in blood serum, liver, and muscle. Before sampling, fish were anesthetized with MS222 (100 mg L^-1^). The blood samples were drawn using 2-mL syringes and collected in 1.5-mL reaction vials. The serum was obtained by centrifugation at 2,000 × *g* at 4°C for 20 min and then stored at -80°C until the assay. Liver and muscle samples were homogenized (1:9 w/v) in a cold (4°C) phosphate buffer solution. The homogenate was centrifuged at 3,000 × *g* for 15 min at 4°C, and then the supernatant was collected for the assay. T-AOC, SOD, LPO, and PC were measured following the manufacturer’s instructions for the total antioxidant capacity assay kit (ABTS method), superoxide dismutase kit, malondialdehyde (MDA) assay kit (TBA method), and protein carbonyl assay kit, respectively (Nanjing Jiancheng Biochemical Corporation, Nanjing, China).

### Texture Measurements and Sensory Evaluation

The texture of muscle was evaluated using a TA-XT Plus Micro TPA device (Stable Micro Systems, Godalming, United Kingdom) equipped with a flat-bottomed cylindrical probe P/36R and a load cell of 250 N. The assay was performed following the method described by Ma et al. (2014) with minor modifications. All texture profiles analysis (TPA) measurements were performed using 2 cm × 2 cm × 2 cm pieces from the dorsal white muscle of each fish. The probe was pressed downward at a constant speed of 1 mm/s into the sample until it reached 60% of the sample height, and the samples were compressed twice for each TPA test. Test conditions were as follows: a pre-test speed of 2 mm/s, a test speed of 1 mm/s, and a post-test speed of 5 mm/s with the stay time of 5 s. Filet heights and the maximum force (g shear force) needed to compress filets to 70% of their height were measured. The TPA and shear force tests were carried out at room temperature; five fish from each pond, with three parallel samples from each fish, were used. Texture curves were generated, and the maximum force was determined as an average of the three measurements.

Sensory analysis of filets was performed by 17 judges who had been trained to test the samples. Eleven sensory indices (color, gloss, texture, springiness, chewiness, sour smell, residues, juiciness, palatability, aroma, acceptability) were judged on a scale from 1 to 9, where 1 indicated no intensity and 9 indicated clear intensity (Table 2).

**Table 2:** Parameters of sensory evaluation of cooked muscle.

Parameter	Score	
White	1: Very weak	9: Intense
Gloss	1: Very weak	9: Intense
Texture	1: Loosely	9: Very firming
Springiness	1: Not elastic	9: Very elastic
Juiciness	1: Dry	9: Very juicy
Palatability	1: Distasteful	9: Very palatable
Aroma	1: Not detected	9: Very fragrant
Chewiness	1: Not chewy	9: Very chewy
Sourish smell	1: Sourish smell	9: Not detected
Residues	1: Many residues	9: Not detected
Acceptability	1: Distasteful	9: Love


### Statistical Analysis

Results are shown as mean with standard deviation. Statistical analyses were performed using SPSS Base 19.0 statistical software (IBM, Armonk, NY, United States). The differences between the control group and experimental group were evaluated by one-way analysis of variance (ANOVA) followed by Duncan’s multiple comparison tests when differences were found using the ANOVA. Statistically significant differences were determined at *P* < 0.05 for all analyses.

## Results

### Growth Performance

The biometric measurements of fish in the experimental ponds differed from those in the control ponds (Table 3). The average weight and multiples of weight gain were significantly higher in the experimental group compared with the control group (*P* < 0.05). There were no significant differences in SL, H, and W between the two groups. Although both CF and visceral mass ratio did not differ significantly between the two groups, the fish had a significantly higher liver somatic index in the control ponds.

**Table 3:** Growth performance of the grass carp from the experimental and control ponds.

	Experimental	Control
Weight (g)	723.06 ± 77.20^*^	641.40 ± 37.40
Length (cm)	35.09 ± 4.66	33.58 ± 0.47
Height (cm)	7.47 ± 1.18	7.27 ± 0.05
Width (cm)	5.23 ± 0.81	5.17 ± 0.05
Multiples of weight gain	12.06 ± 0.62^*^	10.69 ± 0.12
Visceral mass ratio (%)	12.21 ± 0.16	12.24 ± 0.90
Liver somatic index (%)	2.19 ± 0.30	2.40 ± 0.01^*^
Condition factor (g/cm^3^)	1.72 ± 0.02	1.70 ± 0.01


*Vales represent means ± SD, ^∗^ indicates significant difference (*P* < 0.05) between the experimental and the control groups.*

### Chemical Composition of Muscle

Table 4 shows the flesh content, chemical composition of muscle, and cooked rate. There was no difference in the content of water, crude ash, and flesh content between the two groups. The fish in the experimental ponds had significantly lower fat content (*P* < 0.01) and higher protein content (*P* < 0.05) than those in the control ponds. A higher cooked rate (76.74%) was observed in the fish from the experimental ponds (*P* < 0.01).

**Table 4:** Composition and cooked rate of muscle in grass carps from experimental and control ponds.

	Water content (%)	Fat (%)	Crude protein (%)	Crude ash (%)	Flesh content (%)	Cooked rate (%)
Experimental	79.66 ± 0.30	1.13 ± 0.06	17.75 ± 0.13^*^	1.09 ± 0.01	63.91 ± 0.23	76.74 ± 1.59^**^
Control	80.12 ± 0.09	1.41 ± 0.06^**^	17.33 ± 0.07	1.10 ± 0.01	63.64 ± 0.09	74.33 ± 0.17


*Vales represent means ± SD, ^∗^ indicates significant difference (*P* < 0.05) and ^∗∗^ indicates greatly significant difference (*P* < 0.01) between the experimental and the control groups.*

### pH and WHC of Muscle

The fish in the experimental ponds had a significantly higher pH level and WHC than the fish in the control ponds (Table 5). Drip loss, stored loss, and initial frozen leakage rate were all significantly lower in fish from the experimental ponds than in fish from the control ponds (*P* < 0.01). As thawing progressed, the difference between the loss of water from muscle from experimental and control pond fish increased sharply, resulting in a significant difference (*P* < 0.01).

**Table 5:** pH and water holding capacity of muscle in grass carps from experimental and control ponds.

	pH	Drip loss (%)	Stored loss (%)	Frozen leakage rate 0 h (%)	Frozen leakage rate 1 h (%)	Frozen leakage rate 2 h (%)
Experimental	6.06 ± 0.04^*^	1.77 ± 0.13	1.07 ± 0.05	1.06 ± 0.01	1.47 ± 0.06	2.10 ± 0.06
Control	5.92 ± 0.04	2.11 ± 0.08^**^	1.33 ± 0.14^**^	1.26 ± 0.13^*^	1.94 ± 0.01^**^	3.04 ± 0.10^**^


*Vales represent means ± SD, ^∗^ indicates significant difference (*P* < 0.05) and ^∗∗^ indicates greatly significant difference (*P* < 0.01) between the experimental and the control groups.*

### Antioxidant Capacity

**FIGURE 4:**
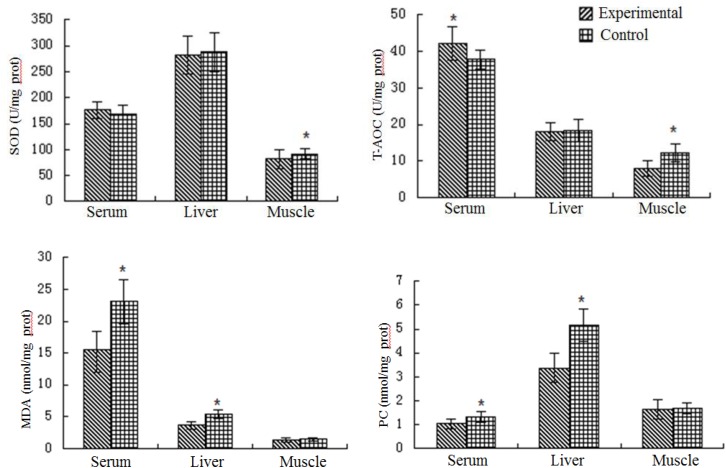
Antioxidant capacity of the grass carp from experimental and control ponds. Significant difference is indicated by ^∗^*P* < 0.05.

The antioxidant capacity in serum, liver, and muscle differed significantly between the two groups (Figure 4). The fish in the experimental ponds had lower SOD activity in muscle and liver compared with fish in the control ponds. Higher T-AOC was detected in the liver of fish in the experimental ponds (*P* < 0.05). The concentrations of MDA and PC were significantly higher in the serum and liver of fish in the control ponds than in the experimental ponds. No significant difference between the two groups in the concentration of either MDA or PC in the muscle of fish was detected.

### Texture Analysis and Sensory Evaluation

**FIGURE 5:**
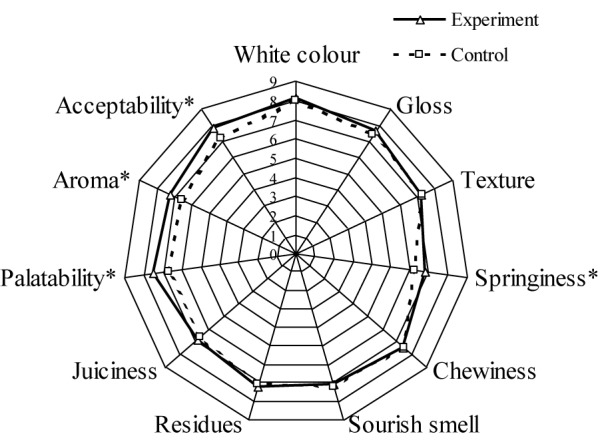
Radar diagram of the average values of sensory properties for the grass carp from experimental and control ponds. Significant difference is indicated by ^∗^*P* < 0.05.

Muscle samples from fish from the experimental group had a higher texture profile (Table 6) and higher sensory evaluation (Figure 5) values than those from the control group. Although samples from the experimental group had consistently higher values of hardness, springiness, gumminess, chewiness, cohesiveness, and resilience compared to the control group, the differences were not statistically significant (Table 6). The sensory evaluation values for springiness, acceptability, aroma, and palatability were significantly higher in the experimental group compared to the control group (Figure 5).

**Table 6:** Texture profile analysis of muscle in grass carps from experimental and control ponds.

	Hardness	Springiness	Cohesiveness	Gumminess	Chewiness	Resilience
Experimental	5269.84 ± 1047.52	0.63 ± 0.06	0.28 ± 0.02	1473.83 ± 325.55	934.53 ± 242.50	0.18 ± 0.03
Control	4767.08 ± 596.97	0.61 ± 0.05	0.29 ± 0.03	1358.57 ± 193.55	832.57 ± 152.99	0.20 ± 0.03


*Vales represent means ± SD.*

## Discussion

In this study, water quality and fish muscle quality were improved by the presence of bio-floating beds in the experimental ponds compared with the control ponds which lacked the beds. Aquatic plants have been widely applied in ecological engineering to improve the quality of surface water and wastewater (Garbett, 2005; Nduvamana et al., 2007). The observed lower concentrations of nitrite, ammonia, and phosphate in the experimental ponds illustrates that water quality was improved significantly by the bio-floating beds in the intensive culture ponds where grass carp were reared in this study. Similar results have been reported in studies of lakes and eutrophic waters (Hu et al., 2008; Li et al., 2010). Aquatic plants assimilate nutrients and create favorable conditions for microbial decomposition of organic matter (Wang et al., 2009). Increases in the nitrogen contents in ponds were probably caused by the accumulation of residual feeds and feces in the sediments, resulting in oxygen consumption. Person-Le et al. (1997) also reported a trend of increasing total nitrogen concentration in an intensive culture pond in the late spring and thereafter. Lower PO_4_^3-^-P and TP concentrations observed in the experimental ponds compared to the control ponds suggested that bio-floating beds could reduce not only nitrogen levels but also phosphorus levels in ponds, supporting the results of other studies (Wen and Recknagel, 2002; Stewart et al., 2008).

Environmental and fish nutritional status are the most important factors that affect fish growth and muscle quality (Johnston, 2001; Larsson et al., 2014). In this study, grass carp cultured in the experimental ponds showed better growth performance in terms of body shape and weight gain than those from control ponds when supplied with the same amounts of nutrients. Water quality is crucial to fish growth (Orban et al., 2002; Björnsson and Ólafsdóttir, 2006), as poor water quality can cause acute or chronic stress and reduce the ability of fish to control homeostasis (Foss et al., 2003; North et al., 2006; Refaey et al., 2017). Thus, the better growth performance of fish in the experimental ponds was attributed primarily to the improved water quality.

The crude protein and fat concentrations of muscle differed between fish from the experimental and control ponds. These differences were probably due to the better water quality in the experimental ponds. Fuentes et al. (2010) reported that wild sea bass (*Dicentrarchus labrax*) had significantly higher protein concentrations compared to farmed fish. Different protein concentration of fish might be caused by different rearing conditions (Haard, 1992). Fish in the experimental ponds also had lower crude fat content than the fish in the control ponds. In general, crude fat content primarily depends on the feed, fish species, and rearing conditions (Love, 1992; Alasalvar et al., 2002; Orban et al., 2002). Lower fat levels in wild fish compared to farmed fish have been observed in some fish species (Alasalvar et al., 2002; Grigorakis et al., 2003; Johnston et al., 2007).

Muscle pH and WHC are important parameters for evaluating muscle quality. Glycolytic potential, fatty acid composition and concentrations, and some biological reactions affect muscle pH (Fernandez et al., 1992), while pH subsequently strongly influences the WHC. A rapid muscle pH decline causes soft texture and poor WHC of the muscle (Ang and Haard, 1985; Anne et al., 2016). On the other hand, high muscle WHC can reduce protein breakdown (Bee et al., 2007). The higher WHC and hardness observed in the experimental fish could be related to higher pH in the muscle, suggesting better muscle quality of grass carp cultured in the ponds with bio-floating beds.

The antioxidant defense system is a highly conserved biochemical mechanism that protects organisms from harmful effects of reactive oxygen species (Birben et al., 2012; Niu et al., 2018). T-AOC is a comprehensive assessment index of antioxidant capacity. The higher T-AOC of grass carp in the experimental ponds indicates that they had greater antioxidant capacity than fish in the control ponds. SOD is an important antioxidant defense in nearly all living cells, as it protects organisms against free radical damage, being the first line of defense in controlling the damaging accumulation of oxygen free radicals (Ramesh et al., 2018). Fish in the control ponds had higher SOD activity than fish in the experimental ponds. This may have been due to greater stress due to the higher nitrite content in the control ponds, which may have activated the antioxidant defense system in the fish to protect themselves (Wang et al., 2004). PC often is used as an indicator of oxidative damage to proteins, and MDA is a product of LPO (Suzuki et al., 2010; Klein et al., 2017). The concentrations of PC and MDA in serum and liver of fish in the experimental ponds were significantly lower than those in the control ponds, which indicates less oxidative damage and LPO in those fish, supporting the idea that the fish cultured in the cleaner water achieved using the bio-floating beds. In addition, liver condition was well from the liver section observation. These results show that fish cultured in a better culture environment had a more effective antioxidative defense system.

Muscle texture is a crucial factor for customer acceptance of and satisfaction with the fish products (Casas et al., 2006; Fuentes et al., 2010). Andersen et al. (1999) reported that higher fat concentration in filets results in less resistance against compression, which indicates a softer texture of fish, and Love (1992) found that lower pH of muscle results in lower hardness in terms of muscle texture. Thus, the greater hardness of muscle in fish from the experimental group may be related to their lower fat content and higher pH. Water content and distribution are additional factors that have a profound influence on muscle texture, such as hardness and juiciness, and a higher WHC may be associated with harder muscle (Offer et al., 1989; Pearce et al., 2011). In this study, muscle from fish in the experimental ponds had a higher WHC and hardness than did muscle from fish in the control ponds. Previous studies demonstrated that muscle from wild-caught fish had higher hardness and springiness, as they inhabited a better water environment compared to cultured fish (Fuentes et al., 2010; Cheng et al., 2014). In this study, the improved water quality in ponds containing bio-floating beds probably affected fish growth, muscle chemical composition, and result in the differences in texture as well. The correlation between water quality and muscle textural quality is speculated that poor water quality will cause chronic stress and reduce fish ability to control homeostasis (North et al., 2006; Refaey et al., 2017), and then may cause the negative effects on muscle growth, such as the size and number of muscle fiber which is reported to determine muscle texture (Taylor et al., 2006; Damez and Clerjon, 2008). In order to confirm this assumption, related experiments need to be done in the future.

In recent years, sensory evaluation with different scores indicating the freshness parameter has been implemented in several European countries (Cheng et al., 2014). In this study, the difference in quality of fish within ponds was generally lower than those among ponds. Fish from the experimental ponds had better scores for springiness, overall acceptability, aroma, and palatability compared to fish from the control ponds. Muscle pH is a vital factor that affects fish muscle appearance and sensory evaluation, with higher pH generally resulting in better scores (Love, 1992; Bee et al., 2007; Luciano et al., 2009). Thus, the better sensory properties of fish from the experimental ponds likely was probably related to their higher muscle pH which, in turn, was brought about by the reduced level of stress experienced by fish in the cleaner water of the experimental ponds.

## Conclusion

This study demonstrated that the presence of bio-floating beds in ponds effectively improved water quality by significantly reducing the concentrations of both nitrite and ammonia in the pond water. The improved culture environment enhanced the growth performance, muscle quality, and antioxidative capacity of the grass carp. Compared with the control ponds, grass carp cultured in the experimental ponds showed better growth performance and had higher crude protein and lower fat concentration in their filets due to the better water quality. In addition, muscle from fish from the experimental ponds had higher WHC, higher antioxidant capacity, and better texture properties such as hardness and chewiness than fish from the control ponds. Fish from the experimental ponds also had better sensory evaluation scores for springiness, acceptability, aroma, and palatability. These results indicate that the use of bio-floating beds in culture ponds improves water quality, fish growth, and muscle quality.

## Author Contributions

JW, DL, and XZ were responsible for the design of the experiments. XZ and JW were responsible for the experiments and data analysis. XZ and DL wrote the manuscript. RT, XH, LL, and YT provides some suggestions and gave the final approval.

## Conflict of Interest Statement

The authors declare that the research was conducted in the absence of any commercial or financial relationships that could be construed as a potential conflict of interest.
